# Impact of car transport availability and drive time on eye examination uptake among adults aged ≥60 years: a record linkage study

**DOI:** 10.1136/bjophthalmol-2018-312201

**Published:** 2018-07-03

**Authors:** David M Wright, Dermot O'Reilly, Augusto Azuara-Blanco, Ruth E Hogg

**Affiliations:** 1 Centre for Public Health, Queen’s University Belfast, Belfast, UK; 2 Administrative Data Research Centre, Northern Ireland, UK

**Keywords:** epidemiology, public health

## Abstract

**Aim:**

To examine associations between uptake of free primary eye care, service availability (density of optometric practices) and service accessibility (household car access and drive time to nearest provider) after accounting for socioeconomic status and other individual, household and area factors.

**Methods:**

We constructed a cohort of 294 870 community-dwelling adults aged 60 years, drawing contextual information from the 2011 Northern Ireland Census. Minimum drive times to the nearest optometry practice (1–19 min) and number of practices were derived for 890 geographical areas. The primary outcome was attendance at one or more publicly funded eye examinations to which all cohort members were entitled between 2009 and 2014. We used multiple log-binomial regression to estimate associations between eye care uptake, car ownership and drive time.

**Results:**

Eye examination uptake was 60.0%. 23.7% of the cohort had no car access, and these individuals had lower uptake than car owners (unadjusted risk ratio (RR) of uptake=0.86 (0.86, 0.87)). Among non-car owners, uptake decreased with drive time (longest vs shortest: RR=0.92 (0.88, 0.97)) with the largest decrease at 4 min drive time (approximately 1.5 miles). This pattern was weaker among car owners. These associations were independent of service availability, which was not associated with uptake.

**Conclusion:**

Both drive time and household car access were associated with eye care use, adjusting for individual, household and area factors. Policies to improve uptake should target those with no car access, especially those beyond walking distance from the nearest eye care provider.

## Introduction

Chronic eye conditions leading to visual impairment significantly reduce quality of life and increase care costs for older people.^1 2^ Progression of leading causes of blindness, wet age-related macular degeneration (AMD), diabetic retinopathy (DR) and glaucoma can be slowed if treated promptly, so early detection is crucial. Regular eye examinations also ensure uncorrected refractive error is addressed, a significant risk factor for occurrence of hip fractures due to falls,^3–5^ themselves associated with high recovery costs.^6 7^ Despite these benefits, uptake of eye care among older people is often lower than recommended by eye care professionals.^8 9^ Barriers to uptake include poor knowledge of the risk of eye diseases and the need for eye examinations and perceived difficulty of visual tests.^10–12^ Individual socioeconomic status (SES) is strongly associated with uptake of eye care where upfront costs are high,^13^ but evidence for this association is equivocal where costs are lower (eg, among older people in the UK who are eligible for free eye examinations and subsidised spectacles).^14^ Associations between eye care uptake and area-level SES have been reported in several contexts,^15 16^ and area deprivation is a risk factor for increased severity of glaucoma and AMD at presentation.^17 18^ Only recently have researchers begun to untangle whether associations stem mainly from the characteristics of individuals or their areas of residence. A multistate survey in the USA indicated that areas with a particular ethnic mix or large proportion of low-income households had higher uptake of eye care, controlling for individual level factors.^15^ The challenge now is to discover how area characteristics influence the individual decisions underpinning access to eye care services.

Uptake of eye care among persons with diabetes, AMD sufferers and the general population has been positively associated at the area level with density of eye care professionals per head of population, a measure of service availability.^15 19 20^ However, it is unknown whether these associations are due to supply limitation (eg, that individuals are unable to get suitable appointment times) or whether there is an additional spatial component; individuals may be discouraged from accessing eye care because they live far from the nearest practitioner. These two dimensions, availability and accessibility, are not necessarily correlated as there may be varying degrees of clustering across areas with identical practitioner density.^21^ Qualitative studies indicate that distance to practitioner is a perceived barrier to eye care uptake among older people,^10^ but there is little quantitative evidence that such perceptions are manifested in terms of behaviour. A descriptive urban study showed that uptake was reduced in areas >15 min walk from the nearest practitioner^22^ but did not account for other area or individual characteristics. Two recent US-based studies estimated drive times to the nearest eye care practitioners for large sections of the population,^23 24^ but the association between these estimates and realised access to eye care has not been quantified. We used records of all publicly funded eye examinations attended by adults aged ≥60 years within Northern Ireland (NI) to investigate whether there were independent associations between uptake of eye care, accessibility and availability of optometrists while accounting for SES and other individual, household and area factors. This age group was chosen because risk of sight-threatening conditions and hence need for surveillance increases with age and because records of eye care uptake across the entire group are available from a single source. We also characterised the relationship between uptake and accessibility to determine whether uptake changes linearly or whether there are key distance thresholds that predict major changes in uptake.

## Methods

### Data sources

In the UK, people aged ≥60 years are eligible for free (publicly funded) eye examinations (biannual for those aged 60–69 years, annual for those ≥70 years, reflecting clinical guidelines). Information on uptake in NI was drawn from the Family Practitioner Services Ophthalmic Database (managed by the Business Services Organisation of the NI Department for Health and Social Care), containing records of all free eye examinations conducted. The database is used to manage payment to service providers and is derived from a partially paper-based administration system. In NI, primary eye care is provided almost exclusively by community optometrists rather than ophthalmologists, and so we refer only to optometrists henceforth. Records of eye examinations of those aged ≥60 years conducted during a 5-year period (October 2009–September 2014 inclusive) were extracted.

### Cohort construction

**Figure 1:**
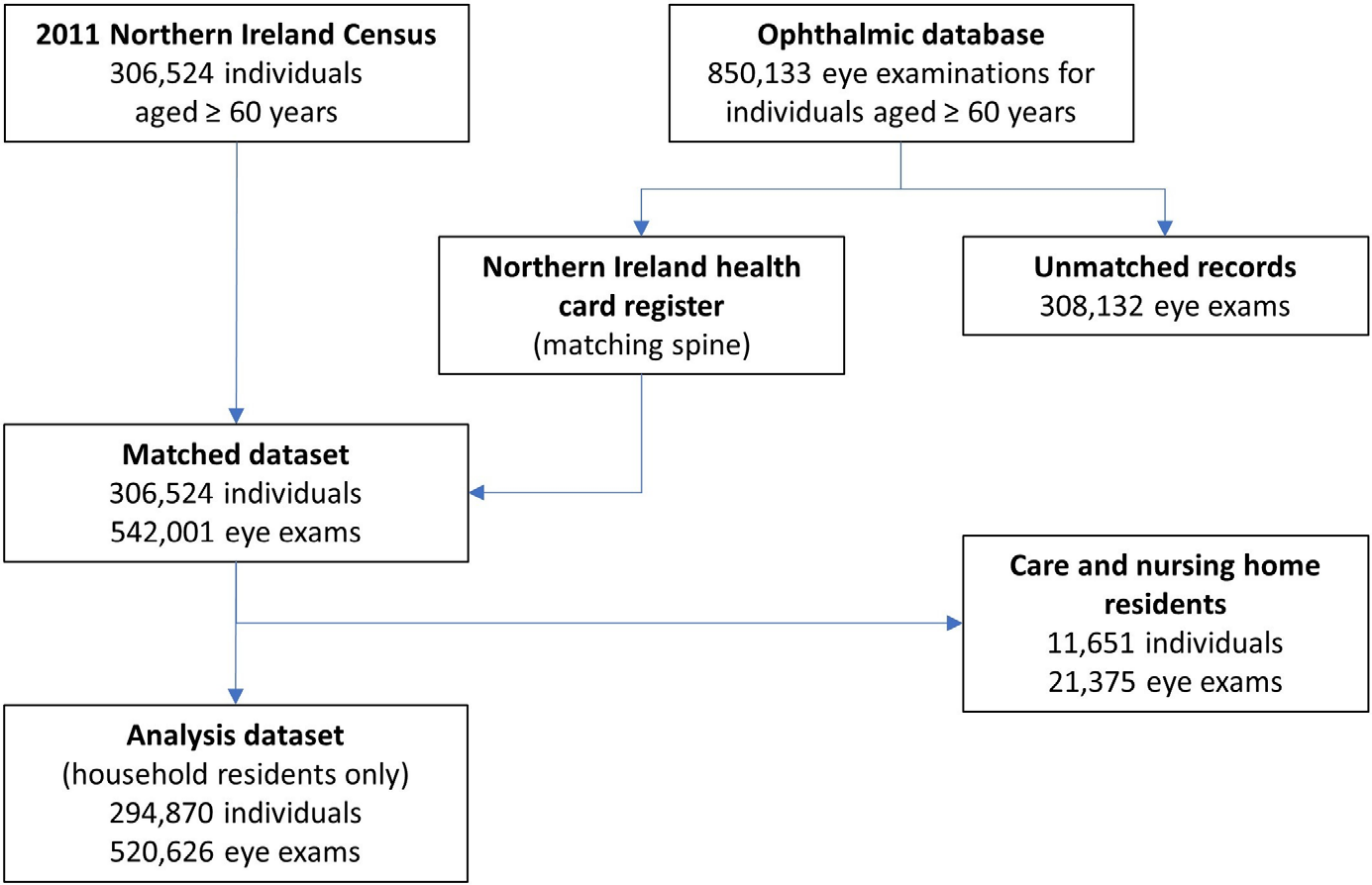
Construction of the linked dataset comprising records from the 2011 Northern Ireland Census and the ophthalmic database.

Cohort construction is summarised in figure 1. Ophthalmic and Census datasets were linked using address information held in the NI health card registration system. Individuals must be registered to access primary healthcare services, and the register contains the current address and also the address history of each individual. Linkages were made using a series of deterministic match keys validated for this type of data (eg, name, address and date of birth). Eye examinations were matched to an individual at any of the addresses at which they had lived during the study period. There were 542 001 eye examination records (63.9% of the total) matched to individuals. Linkages were made within the Administrative Data Research Centre-NI. To protect individual privacy, records were deidentified before the researchers accessed the linked dataset. Ethical approval for the study was received from the UK National Research Ethics Service (reference: 16/EM/0103).

The analysis cohort consisted of all community-dwelling respondents to the 2011 Census aged ≥60 years at the beginning of the study period. Those in care and nursing homes (n=11 651) were excluded as social factors influencing this group are likely to differ from those affecting community dwellers. The cohort consisted of 294 870 individuals from 215 557 households; 263 568 cohort members (89.4%) survived follow-up.

### Uptake, accessibility and availability measures

The outcome variable was a binary indicator of any uptake during the study period (ie, had attended at least one eye examination). Accessibility of eye care was measured as drive time to the nearest registered optometry practice and density of practices was used to measure availability. Drive times and practice densities were calculated for 890 Super Output Areas (SOAs), administrative units with average population 2000 (total population of NI at the Census=1 810 863). The SOA geography was designed to improve the reporting of small area statistics from Census results by ensuring that SOAs had approximately equal population sizes and that boundaries were not altered over time to allowing meaningful analysis of population trends. Density per 1000 head of population was calculated by SOA using a published list of registered practices.

Drive times to the nearest practice were derived from modelled estimates, part of a set of area deprivation measures produced by the Northern Ireland Statistics and Research Agency.^25^ The modelling exercise used the MapPoint Europe software (Microsoft, 2002) to estimate minimum drive times between area centroids based on a map of the road network with speeds assigned by road type. Drive times from each area of residence to the nearest area containing a practice are published for a fine administrative geography (Output Areas, n=5022, average population: 300). These were aggregated by SOA so that the linked dataset would comply with data provider rules to prevent inadvertent disclosure of personal information. The effect of aggregation was to marginally reduce variability in drive times in the analysis dataset. Each SOA was assigned the mean drive time of the component Output Areas, and drive times were categorised prior to modelling so each category had sufficient data support. Travel times by other modes of transport were not considered because car transport is the dominant mode in NI. The rail network links only the major towns, and bus services account for a small proportion of journeys. Recent estimates show that among those aged ≥60 years, 80% of journeys by men and 78% of journeys by women were made using car or taxi.^26^ The remainder were predominantly made on foot (16% and 17%, respectively). Independent walking and cycling infrastructure is not well developed and so walking and cycling travel times are likely to be highly correlated with drive times (although longer).

### Other variables

Age, sex, ethnicity, religion, highest educational attainment, self-reported health status and caregiving status were drawn from 2011 NI Census returns. At the household level, housing tenure, car access and whether accommodation was adapted for visual impairment were selected along with a classification of household structure (eg, living alone/living with a partner). An area measure of SES was also selected, the proportion of the population in households receiving income-related benefits or tax credits.^25^

### Statistical analysis

Models were fitted to estimate associations between predictor variables and uptake. The event of interest was any uptake during the study period, indicated for each individual by linkage to one or more eye examination records. As this event was relatively common, log-binomial models were used as they give unbiased estimates of associations in this situation (logistic models are severely biased when the outcome event occurs in >10% of cases). Risk of uptake was compared among levels using risk ratios (RRs). Uptake was modelled as a function of each variable singly followed by a multivariable model containing main effects for all variables (ie, simultaneously adjusting for individual, household and area level variables). An interaction term was included between the variables of primary interest, drive time and household car access to determine the extent to which these factors combine to influence uptake while adjusting for all other variables. Interaction terms were displayed using effect plots^27^ on the probability scale. Time ‘at risk’ (ie, proportion of the study period survived) was included in multivariable models to account for differential survival of cohort members. All models were fitted using *R* software.^28^

### Sensitivity analyses

We conducted two sensitivity analyses: the first assessing the ways in which inclusion of domiciliary eye examinations modified our estimates and the second assessing the influence of imperfect matching of the two datasets on the estimated associations. Full details of these two analyses are given in online supplementary material 1 and online supplementary figure 1.

10.1136/bjophthalmol-2018-312201.supp2 Supplementary data

10.1136/bjophthalmol-2018-312201.supp1 Supplementary data

## Results

A total of 177 111 cohort members (60.0%) attended at least one eye exam during the 5-year study period. Table 1 shows individual and household characteristics of the cohort and associations between these variables and uptake. Educational attainment and housing tenure are included as key measures of SES. Of the other variables, only those most strongly associated with uptake (relative risk differentials>5% between at least two variable levels in the fully adjusted model) and with all levels containing >0.1% of the cohort are displayed (estimates for other variables available on request). Uptake was lower among men, and there was an inverse U-shaped relationship between uptake and age, peaking between ages 70 and 75 years. Those with cognitive difficulties had substantially lower uptake, but presence of physical health conditions was not strongly associated with uptake. Most of these relative risk differentials were attenuated following adjustment for other variables but remained statistically significant. Prior to adjustment, there were modest gradients in uptake with measures of SES; those with no qualifications had lower uptake than degree holders and renters were less likely to attend than homeowners. However, these differentials virtually disappeared following adjustment.

**Table 1:** Individual and household characteristics and associations between characteristics and uptake of free eye examinations, 2009–2014, among those aged ≥60 years in Northern Ireland, UK

Variable	Level	Total (N=2 94 870) (%)	Attended eye examination (%)	Single variable model RR (95% CI)	Adjusted model RR (95% CI)*
Age (years)	(60,65)	29.2	59.9	1.00	1.00
	(65,70)	23.8	62.9	1.05 (1.04 to 1.06)	1.04 (1.04 to 1.05)
	(70,75)	18.9	63.8	1.06 (1.06 to 1.07)	1.07 (1.06 to 1.08)
	(75,80)	14.0	61.5	1.03 (1.02 to 1.04)	1.06 (1.05 to 1.07)
	(80,85)	8.8	54.3	0.91 (0.89 to 0.92)	0.98 (0.97 to 0.99)
	(85,90)	4.2	44.2	0.74 (0.72 to 0.75)	0.83 (0.82 to 0.85)
	(90,120)	1.2	31.1	0.52 (0.49 to 0.54)	0.62 (0.59 to 0.65)
Sex	Female	55.0	61.3	1.00	1.00
	Male	45.0	58.6	0.96 (0.95 to 0.96)	0.93 (0.92 to 0.94)
Religion	Protestant and other Christian (including Christian related)	63.2	62.1	1.00	1.00
	Catholic	34.8	56.5	0.91 (0.90 to 0.92)	0.93 (0.92 to 0.93)
	No religion	1.2	57.1	0.92 (0.89 to 0.95)	0.97 (0.94 to 0.99)
	Other religions	0.7	61.8	0.99 (0.96 to 1.03)	1.02 (0.99 to 1.06)
Chronic health conditions	Blind	5.7	54.1	0.90 (0.88 to 0.91)	0.99 (0.97 to 1.00)
	Communication difficulties	1.9	40.0	0.66 (0.64 to 0.68)	0.82 (0.80 to 0.85)
	Chronic illness	20.8	61.9	1.04 (1.03 to 1.05)	1.06 (1.05 to 1.07)
	Breathing difficulty	17.9	60.9	1.02 (1.01 to 1.02)	1.05 (1.04 to 1.06)
	Deaf	18.8	60.4	1.01 (1.00 to 1.01)	1.06 (1.05 to 1.07)
	Learning difficulty	0.7	42.3	0.70 (0.67 to 0.74)	0.89 (0.84 to 0.93)
	Chronic pain	27.3	60.5	1.01 (1.00 to 1.02)	1.04 (1.03 to 1.05)
	Mobility difficulty	35.1	58.0	0.95 (0.94 to 0.95)	1.00 (0.99 to 1.01)
	Mental condition	5.5	56.1	0.93 (0.92 to 0.94)	0.99 (0.98 to 1.01)
	Memory loss	5.1	43.8	0.72 (0.71 to 0.73)	0.83 (0.82 to 0.85)
	Other condition	9.5	60.1	1.00 (0.99 to 1.01)	1.02 (1.01 to 1.03)
Educational attainment	Degree (bachelor’s or higher)	15.2	63.0	1.00	1.00
	Two or more A-levels	3.4	62.1	0.99 (0.97 to 1.00)	0.99 (0.97 to 1.00)
	Five or more GCSEs	6.4	63.6	1.01 (1.00 to 1.02)	1.00 (0.99 to 1.01)
	Apprenticeship	5.0	62.0	0.98 (0.97 to 1.00)	1.01 (0.99 to 1.02)
	Vocational/other	5.1	60.5	0.96 (0.95 to 0.97)	0.98 (0.96 to 0.99)
	Foundation	4.5	62.1	0.99 (0.97 to 1.00)	0.98 (0.97 to 1.00)
	No qualifications	60.3	58.5	0.93 (0.92 to 0.93)	0.97 (0.97 to 0.98)
Housing tenure	Owner occupied	77.3	61.0	1.00	1.00
	Private rented	5.3	55.9	0.92 (0.90 to 0.93)	0.96 (0.95 to 0.97)
	Social rented	13.5	57.5	0.94 (0.93 to 0.95)	1.01 (1.00 to 1.02)
	Rent free	4.0	56.9	0.93 (0.92 to 0.95)	1.01 (0.99 to 1.02)
Car access†	Yes	76.3	62.1	1.00	
	No	23.7	53.6	0.86 (0.86 to 0.87)	
Household structure	Alone	29.0	58.0	1.00	1.00
	Partner only	43.1	64.2	1.11 (1.10 to 1.11)	1.03 (1.02 to 1.03)
	Partner and child(ren)	13.0	59.4	1.02 (1.01 to 1.03)	0.97 (0.96 to 0.98)
	Partner and others	1.1	60.3	1.04 (1.01 to 1.07)	0.98 (0.95 to 1.00)
	Children only	6.1	56.1	0.97 (0.95 to 0.98)	0.96 (0.95 to 0.97)
	Siblings	2.2	45.6	0.79 (0.76 to 0.81)	0.79 (0.77 to 0.81)
	Complex/other	5.6	50.5	0.87 (0.86 to 0.88)	0.87 (0.86 to 0.89)
Accommodation adapted for visual impairment	No	99.6	60.1	1.00	1.00
	Yes	0.4	50.9	0.85 (0.8 to 0.89)	0.93 (0.89 to 0.98)

a*Adjusted for all other variables (including those in table 2) and ethnicity, general health, caregiving, area income deprivation, density of optometry practices, drive time to nearest practice and time at risk (years of study period survived).

†Adjusted RR not shown as model included interaction between car access and time to nearest optometry practice.

GCSE, General Certificate of Secondary Education; RR, risk ratio.

**Table 2:** Area characteristics and associations between characteristics and uptake of free eye examinations, 2009 to 2014, among those aged ≥60 years in Northern Ireland, UK

Variable	Level	Total (N=2 94 870) (%)	Attended eye examination (%)	Single variable model RR (95% CI)	Adjusted model RR (95% CI)*
Density of optometry practices (per 1000 people)	0	80.2	60.2	1.00	1.00
	(0, 1)	15.1	59.6	0.99 (0.98 to 1.00)	0.99 (0.98 to 1.00)
	(1, 4.57)	4.7	58.9	0.98 (0.96 to 0.99)	0.99 (0.97 to 1.00)
Drive time to nearest optometry practice (minutes)†	(0, 2)	46.5	60.6	1.00	
	(2, 4)	24.5	61.6	1.02 (1.01 to 1.02)	
	(4, 6)	11.9	58.8	0.97 (0.96 to 0.98)	
	(6, 8)	7.7	57.8	0.95 (0.94 to 0.96)	
	(8, 10)	5.1	56.9	0.94 (0.92 to 0.95)	
	(10, 20)	4.4	56.4	0.93 (0.92 to 0.95)	
Area income deprivation (quintiles)	1 (least deprived)	20.5	63.3	1.00	1.00
	2	22.1	59.8	0.94 (0.94 to 0.95)	0.98 (0.97 to 0.98)
	3	17.3	59.0	0.93 (0.92 to 0.94)	0.98 (0.97 to 0.99)
	4	20.5	58.6	0.93 (0.92 to 0.93)	0.98 (0.97 to 0.98)
	5 (most deprived)	19.6	59.5	0.94 (0.93 to 0.95)	1.01 (1.00 to 1.02)

*Adjusted for all other variables (including those in table 1) and ethnicity, general health, caregiving, area income deprivation, density of optometry practices, drive time to nearest practice and time at risk (years of study period survived).

†Adjusted RR not shown as model included interaction between car access and time to nearest optometry practice.

RR, risk ratio; CI, Confidence Interval. ^a^ Adjusted for all other variables (including those in table 1) and ethnicity, general health, caregiving, area income deprivation, density of optometry practices, drive time to nearest practice and time at risk (years of study period survived). ^b^ Adjusted RR not shown as model included interaction between car access and time to nearest optometry practice.

RR, risk ratio.

RR, risk ratio.

### Drive times and density of optometrists

Table 2 shows associations between area characteristics and eye exam uptake. There was little variation among areas in density of optometry practices (80% of areas had none), and we found no evidence that uptake was associated with practice density or area deprivation. Uptake was negatively associated with drive time to the nearest practice. The majority (71%) of the cohort had drive times<4 min. In the single variable model, those with the longest drive times (>10 min) were 7% less likely (RR=0.93 (0.92, 0.95)) to attend relative to those with the shortest (<2 min).

### Car access and drive times

**Figure 2:**
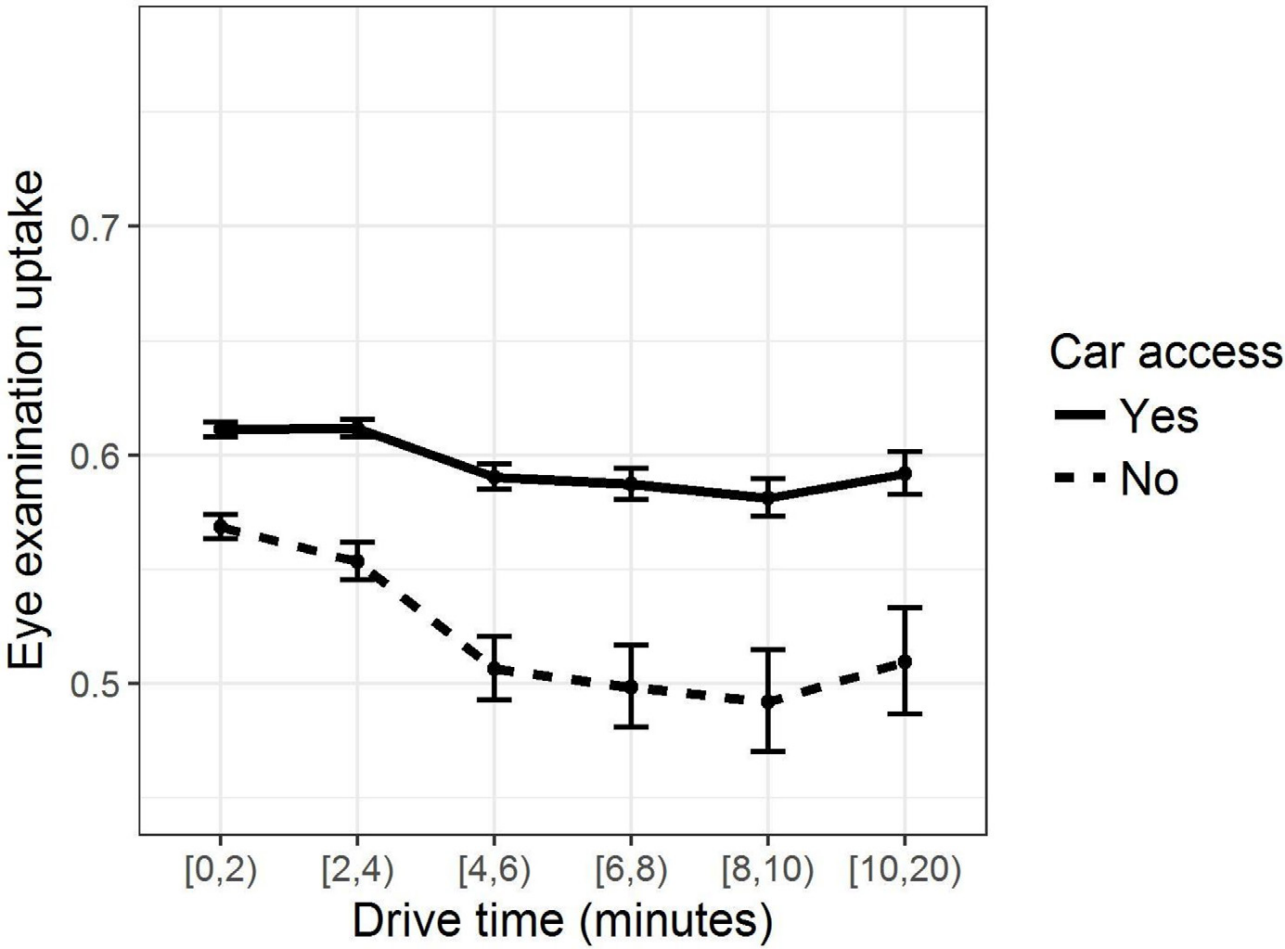
Estimated uptake of eye examinations, 2009–2014, by household car access and drive time to the nearest optometry practice, Northern Ireland, UK. Point estimates (proportions) and 95% CIs given.

Those without household car access comprised almost a quarter of the cohort and in the single variable model were an estimated 14% less likely (RR=0.86 (0.86, 0.87]) to have attended an eye exam relative to car owners (table 1). The relationship between drive time and uptake differed between those with and without cars (figure 2). Uptake was highest among car owners with drive times of less than 4 min. There was a negligible decrease in uptake (RR=0.97 (0.96, 0.98)) at 4 min but little evidence for further change with increasing drive time. Among non-car owners, the decrease in uptake at 4 min was more pronounced (RR=0.92 (0.89, 0.95)), and the difference in uptake between the highest and the lowest drive times was proportionally much larger (RRs: car owners=0.97 (0.95, 0.98); non-car owners=0.92 (0.88, 0.97)).

## Discussion

### Accessibility barriers to eye care uptake

In this population-wide study, we identify accessibility as a significant barrier to uptake of routine eye care among those aged ≥60 years, independent of service availability and individual, household and area factors. This is a novel finding. Geography influences health-seeking behaviour for non-ocular conditions,^21 29 30^ and here we demonstrate the importance of accessibility as well as availability for eye care uptake.

Three key findings emerged; first that uptake of eye examinations was substantially lower among those without car access. Public transport or lifts from others appear less favourable means of transport for those accessing routine eye care. This is in line with general travel preferences for this age group^26^ and despite the fact that all those in our cohort are eligible for free public transport. Second, the greatest decrease in uptake for non-car owners occurred when drive times exceeded 4 min, a distance of approximately 1.5 miles. This may indicate the maximum distance those aged ≥60 years will walk to access eye care. Third, uptake was largely independent of both individual and household SES (in terms of educational attainment and housing tenure) and service availability.

Car ownership levels are high in most developed countries including the UK, and this may explain why survey-based studies have identified other factors, principally low perceived need and financial constraints, as more prominent barriers to eye care uptake than service accessibility.^8 10 12^ However, car ownership decreases with age, and the proportion reporting transport problems as the main barrier to uptake substantially increases with age.^9^ Therefore, ensuring accessibility of eye care is likely to be increasingly important as populations age.

We found little variation in eye care uptake among car owners, but the observed range of drive times was narrow. The maximum drive time in this study (19 min) was less than the average drive time among patients travelling to receive healthcare in the USA,^28^ and drive times to access eye care in some areas are likely to be longer given the clustering of eye care professionals.^30^ Should similar data be available for an area with greater distances to eye care, it would be important to confirm the current findings and determine whether a time threshold exists beyond which car owners are discouraged from accessing eye care.

### Strengths and limitations

The main strength is the large linked dataset, the Census providing comprehensive coverage (>95% response rate for this age group) and rich contextual information on a population of almost 300 000. The number of eye examination records used was an order of magnitude greater than in the largest previous study of realised uptake.^16^ The ophthalmic database contained information on all publicly funded eye exams, enabling us to focus on uptake of standard services but account for domiciliary services that reduce demand for standard services in some groups. The use of data collected for administrative rather than research purposes has the limitation that potential confounders may be absent from the available datasets. In this study, adjustment for presence of ocular or ocular-linked systemic conditions (eg, glaucoma and diabetes) would have been desirable, but this information was not collected in the Census. Similarly, we had no information on privately funded eye examinations, records of which are held by individual practices and are not available for research. However, given the universal eligibility for free examinations in this age group, private tests are rare so our estimates are unlikely to be biased substantially. A more severe limitation concerns the large proportion of eye exams not matched to Census records. Matching failed when the primary patient identifier (a unique number used across the healthcare system) was incorrect in ophthalmic records, either because incorrect information was provided by the patient or because errors were introduced during digitisation of paper-based records. Our main findings were robust to the influence of matching failure under the assumption that non-matching was a random process. This assumption is plausible for digitisation errors, but some individual characteristics (eg, very old age, cognitive impairment) may be associated with increased likelihood of providing incorrect information. The magnitude of any bias introduced by this mechanism is unknown, but adjustment for the full range of individual characteristics is likely to have mitigated the problem.

### Implications

Variation in eye examination uptake with drive time and between car owners and non-car owners was modest, but the size of the groups affected means that these gradients have important public health implications. Almost 70 000 people did not have car access, and given the observed RRs, this equates to approximately 6000 fewer people using eye care services than if car access had been universal. Public health interventions might reduce the influence of poor accessibility on eye care uptake. Most basic would be advertising in the least accessible areas to highlight the importance of regular eye examinations. Such campaigns have been shown to increase use of eye care services among the over 70s.^31^ Where electronic eye exam records exist, they may be used both to target and assess the effectiveness of interventions, with personalised reminders sent to those with irregular attendance. Directly increasing accessibility through travel subsidies or arranged transport would be more costly as would provision of mobile eye exam clinics, similar to those used in the UK for DR screening. Mobile clinics are often colocated with other primary healthcare providers, which are typically closer to residential areas than optometry practices.^32^ Focus groups indicate that older people would prefer primary care services to be grouped this way.^10^ Finally, domiciliary services might be extended to those unwilling to travel rather than those physically unable. A disadvantage is that elements of the standard eye exam are more difficult to conduct in the home, potentially leaving sight-threatening conditions undiagnosed. Recent evidence suggests that even under standard (non-domiciliary) eye examination, AMD is underdiagnosed by primary care practitioners.^33^ However, advances in portable ophthalmoscope technology^34^ or mobile phone-based vision self-testing may reduce the need for eye examinations to be conducted in optometry practices. If so, unwillingness or inability to travel need not be a barrier to eye care access in future.

More broadly, our findings may be useful to both eye specialists and other healthcare professionals seeking to increase uptake of primary eye care. At other points of contact with health services, Individuals with characteristics strongly associated with low uptake (eg, aged over 70 years, males) could be asked when their last eye examination was and reminded of the importance of regular examinations.

## Conclusion

Using a large population scale linked administrative dataset, we show that accessibility plays an important role in determining uptake of routine eye care independent of service availability or individual characteristics. Policies to improve uptake would be best targeted towards those without household car access and especially those living beyond walking distance from the nearest eye care provider.

## References

[1] Crews JE, Chou C-F, Zhang X, et al Health-related quality of life among people aged ≥65 years with self-reported visual impairment: findings from the 2006–2010 behavioral risk factor surveillance system. Ophthalmic Epidemiol 2014;21:287–96. 10.3109/09286586.2014.926556 24955821 PMC4924345

[2] Köberlein J, Beifus K, Schaffert C, et al The economic burden of visual impairment and blindness: a systematic review. BMJ Open 2013;3:e003471 10.1136/bmjopen-2013-003471 PMC3822298 24202057

[3] Loriaut P, Loriaut P, Boyer P, et al Visual impairment and hip fractures: a case-control study in elderly patients. Ophthalmic Res 2014;52:212–6. 10.1159/000362881 25378036

[4] Lord SR Visual risk factors for falls in older people. Age Ageing 2006;35(Suppl 2):ii42–5. 10.1093/ageing/afl085 16926203

[5] Cox A, Blaikie A, Macewen CJ, et al Optometric and ophthalmic contact in elderly hip fracture patients with visual impairment. Ophthalmic Physiol Opt 2005;25:357–62. 10.1111/j.1475-1313.2005.00307.x 15953121

[6] Burns ER, Stevens JA, Lee R The direct costs of fatal and non-fatal falls among older adults - United States. J Safety Res 2016;58:99–103. 10.1016/j.jsr.2016.05.001 27620939 PMC6823838

[7] Hartholt KA, Polinder S, Van der Cammen TJ, et al Costs of falls in an ageing population: a nationwide study from the Netherlands (2007-2009). Injury 2012;43:1199–203. 10.1016/j.injury.2012.03.033 22541759

[8] Chou CF, Sherrod CE, Zhang X, et al Barriers to eye care among people aged 40 years and older with diagnosed diabetes, 2006-2010. Diabetes Care 2014;37:180–8. 10.2337/dc13-1507 24009300 PMC4930070

[9] Conway L, McLaughlan B Older people and eye tests:don’t let age rob you of your sight. 2007.

[10] Shickle D, Griffin M Why don't older adults in England go to have their eyes examined? Ophthalmic Physiol Opt 2014;34:38–45. 10.1111/opo.12100 24325433

[11] Biddyr S, Jones A Preventing sight loss in older people. A qualitative study exploring barriers to the uptake of regular sight tests of older people living in socially deprived communities in South Wales. Public Health 2015;129:110–6. 10.1016/j.puhe.2014.10.013 25687709

[12] Leamon S, Hayden C, Lee H, et al Improving access to optometry services for people at risk of preventable sight loss: a qualitative study in five UK locations. J Public Health 2014;36:667–73. 10.1093/pubmed/fdt130 PMC4245897 24408903

[13] Zhang X, Cotch MF, Ryskulova A, et al Vision health disparities in the United States by race/ethnicity, education, and economic status: findings from two nationally representative surveys. Am J Ophthalmol 2012;154(Suppl):S53–62. 10.1016/j.ajo.2011.08.045 23158224 PMC4169111

[14] Knight A, Lindfield R The relationship between socio-economic status and access to eye health services in the UK: a systematic review. Public Health 2015;129:94–102. 10.1016/j.puhe.2014.10.011 25682906

[15] Chou CF, Beckles GL, Cheng YJ, et al Association between county-level characteristics and eye care use by US Adults in 22 states after accounting for individual-level characteristics using a conceptual framework. JAMA Ophthalmol 2016;134:1158–67. 10.1001/jamaophthalmol.2016.3007 27561117

[16] Shickle D, Farragher TM Geographical inequalities in uptake of NHS-funded eye examinations: small area analysis of Leeds, UK. J Public Health 2015;37:337–45. 10.1093/pubmed/fdu039 25015580

[17] Ng WS, Agarwal PK, Sidiki S, et al The effect of socio-economic deprivation on severity of glaucoma at presentation. Br J Ophthalmol 2010;94:85–7. 10.1136/bjo.2008.153312 19628488

[18] Yip JL, Khawaja AP, Chan MP, et al Area deprivation and age related macular degeneration in the EPIC-Norfolk eye study. Public Health 2015;129:103–9. 10.1016/j.puhe.2014.10.012 25687711 PMC4357435

[19] Chou CF, Zhang X, Crews JE, et al Impact of geographic density of eye care professionals on eye care among adults with diabetes. Ophthalmic Epidemiol 2012;19:340–9. 10.3109/09286586.2012.722244 23088291

[20] Gibson DM Eye care availability and access among individuals with diabetes, diabetic retinopathy, or age-related macular degeneration. JAMA Ophthalmol 2014;132:471–7. 10.1001/jamaophthalmol.2013.7682 24458097

[21] Guagliardo MF Spatial accessibility of primary care: concepts, methods and challenges. Int J Health Geogr 2004;3:3 10.1186/1476-072X-3-3 14987337 PMC394340

[22] Public Health Action Support Team. Care needs assessment: eye health findings and recommendations. 2009.

[23] Stein JD, Kapoor KG, Tootoo JL, et al Access to ophthalmologists in states where optometrists have expanded scope of practice. JAMA Ophthalmol 2018;136:39-45 10.1001/jamaophthalmol.2017.5081 29167903 PMC5833600

[24] Lee CS, Morris A, Van Gelder RN, et al Evaluating access to eye care in the contiguous United States by calculated driving time in the united states medicare population. Ophthalmology 2016;123:2456–61. 10.1016/j.ophtha.2016.08.015 27633646 PMC5608548

[25] NISRA. Northern Ireland multiple deprivation measure 2010. 2010.

[26] NISRA. Travel survey for Northern Ireland in-depth report 2011-2013. 2014.

[27] Fox J Effect displays in R for generalised linear models. J Stat Softw 2003;8:1–27. 10.18637/jss.v008.i15

[28] R Development Core Team. R: a language and environment for statistical computing. Vienna, Austria: R Foundation for Statistical Computing, 2016.

[29] Vallée J, Chauvin P Investigating the effects of medical density on health-seeking behaviours using a multiscale approach to residential and activity spaces: results from a prospective cohort study in the Paris metropolitan area, France. Int J Health Geogr 2012;11:54 10.1186/1476-072X-11-54 23268832 PMC3554434

[30] Probst JC, Laditka SB, Wang JY, et al Effects of residence and race on burden of travel for care: cross sectional analysis of the 2001 US National Household Travel Survey. BMC Health Serv Res 2007;7:40 10.1186/1472-6963-7-40 17349050 PMC1851736

[31] Müller A, Keeffe JE, Taylor HR Changes in eye care utilization following an eye health promotion campaign. Clin Exp Ophthalmol 2007;35:305–9. 10.1111/j.1442-9071.2007.01450.x 17539780

[32] Low L, O'Colmain U, Ogston S, et al Accessibility of high-street optometry premises within Tayside. Br J Ophthalmol 2013;97:1216–7. 10.1136/bjophthalmol-2013-303471 23775403

[33] Neely DC, Bray KJ, Huisingh CE, et al Prevalence of undiagnosed age-related macular degeneration in primary eye care. JAMA Ophthalmol 2017;135:570–5. 10.1001/jamaophthalmol.2017.0830 28448669 PMC5847085

[34] Bastawrous A, Giardini ME, Bolster NM, et al Clinical validation of a smartphone-based adapter for optic disc imaging in Kenya. JAMA Ophthalmol 2016;134:151–8. 10.1001/jamaophthalmol.2015.4625 26606110 PMC5321504

