# Supplementary material for: Impact of car transport availability and drive time on eye examination uptake among adults aged ≥60 years: a record linkage study
Source: Br J Ophthalmol. 2018 Jul 3;103(6):730–6. doi: 10.1136/bjophthalmol-2018-312201 (PMC6582726; doi:10.1136/bjophthalmol-2018-312201)
Supplement: Supplementary data [file bjophthalmol-2018-312201supp002.pdf]

Impact of car transport availability and drive time on eye examination uptake among adults aged ≥60 years: a record linkage study

David M. Wright, Dermot O'Reilly, Augusto Azuara-Blanco, Ruth E. Hogg

Supplementary material – sensitivity analyses

Domiciliary examinations

Those whose health precludes travel to an optometry practice are eligible to receive domiciliary eye examinations. Cohort members received a total of 17,895 domiciliary exams during the study period. We reran the adjusted model using a revised outcome measure, uptake of either a standard or domiciliary exam, to assess whether patterns of association differed when domiciliary exams were included (i.e. was low uptake of standard tests for some groups offset by high uptake of domiciliary exams).

There was no change in the pattern of associations between drive time and uptake when domiciliary exams were included in the analysis. Uptake increased across all groups indicating that some cohort members had domiciliary exams only and the differential in uptake between car owners and non-car owners narrowed (Figure S1), indicating that non-car owners were disproportionately more likely to access domiciliary eye exams. Differentials in uptake among age groups were attenuated when domiciliary exams were included, indicating that the low uptake of standard exams among the oldest age groups was balanced by increased use of domiciliary services (compare Table 1 with Table S1). A similar pattern was observed for those with memory loss or communication difficulties. There were no substantial differences in the pattern of associations between uptake and any of the other individual or household characteristics following inclusion of domiciliary exams.

Imperfect matching

A sensitivity analysis of the influence of imperfect matching of the two datasets on estimated associations was conducted using multiple imputation. Two hundred imputed datasets were generated and in each instance unmatched exams were imputed to cohort members, assuming that non-matching was the result of a random process not associated with the measured covariates (e.g. administrative error). The outcome variable was recalculated to include the imputed exams; uptake status was changed following imputation for those without matched exams but was unchanged for those with an exam history. Multiple exams could be imputed to the same individual, subject to the constraint that there must be an interval of at least a year between the imputed exam and all other

exams. Adjusted models were rerun for each imputation and distribution of parameters and model predictions across imputations were informally compared with those estimated in the main analysis.

When the non-matched eye exam records were imputed, estimated uptake increased by approximately 15% in absolute terms but the pattern of associations between uptake, drive time and car access remained similar. There was a clear separation between car owners and non-car owners, a slight decrease in uptake with drive time for car owners and a more pronounced decrease at four minutes for non-car owners (Figure S1). However, variation both between and within groups was reduced with estimates tending towards the mean.

Table S1. Associations between individual and household characteristics and uptake of free eye examinations, 2009 to 2014, among those aged ≥60 years and older in Northern Ireland, UK. Examinations conducted both in optometry practices and in the home (domiciliary examinations) are included.

**Variable**	**Level**	**Adjusted model RR (95% CI)[a]**
Age	[60,65)	1.00
	[65,70)	1.05 (1.04, 1.06)
	[70,75)	1.09 (1.08, 1.10)
	[75,80)	1.11 (1.10, 1.12)
	[80,85)	1.10 (1.09, 1.11)
	[85,90)	1.06 (1.04, 1.08)
	[90,120)	0.98 (0.96, 1.01)
Sex	Female	1.00
	Male	0.93 (0.92, 0.94)
Religion	Protestant and Other Christian (including Christian Related)	1.00
	Catholic	0.92 (0.91, 0.93)
	No religion	0.97 (0.95, 0.99)
	Other religions	1.01 (0.98, 1.04)
Chronic health conditions	Blind	0.99 (0.98, 1.00)
	Communication difficulties	0.91 (0.89, 0.93)
	Chronic illness	1.05 (1.04, 1.06)
	Breathing difficulty	1.03 (1.02, 1.03)
	Deaf	1.05 (1.04, 1.06)
	Learning difficulty	0.91 (0.88, 0.95)
	Chronic pain	1.03 (1.02, 1.04)
	Mobility difficulty	1.02 (1.01, 1.03)
	Mental condition	0.99 (0.98, 1.01)
	Memory loss	0.97 (0.95, 0.98)
	Other condition	1.02 (1.01, 1.03)
Educational attainment	Degree	1.00
	Two or more A-levels	0.98 (0.97, 1.00)
	Five or more GCSEs	1.00 (0.99, 1.01)
	Apprenticeship	1.01 (0.99, 1.02)
	Vocational/other	0.98 (0.97, 0.99)
	Foundation	0.98 (0.97, 1.00)
	No qualifications	0.97 (0.96, 0.98)
Housing tenure	Owner occupied	1.00
	Private rented	0.96 (0.95, 0.97)
	Social rented	1.03 (1.02, 1.04)
	Rent free	1.02 (1.00, 1.03)

Household structure	Alone	1.00
	Partner only	1.01 (1.01, 1.02)
	Partner and child(ren)	0.96 (0.95, 0.97)
	Partner and others	0.96 (0.94, 0.99)
	Children only	0.95 (0.94, 0.96)
	Siblings	0.81 (0.79, 0.82)
	Complex/other	0.87 (0.86, 0.88)
Accommodation adapted for visual impairment	No	1.00
	Yes	0.96 (0.92, 1.01)

[a] Adjusted for all other variables and ethnicity, general health, caregiving, area income deprivation, density of optometry practices, drive time to nearest practice and time at risk (years of study period survived).
