# Supplementary figures and images for: Impact of car transport availability and drive time on eye examination uptake among adults aged ≥60 years: a record linkage study
Source: Br J Ophthalmol. 2018 Jul 3;103(6):730–6. doi: 10.1136/bjophthalmol-2018-312201 (PMC6582726; doi:10.1136/bjophthalmol-2018-312201)

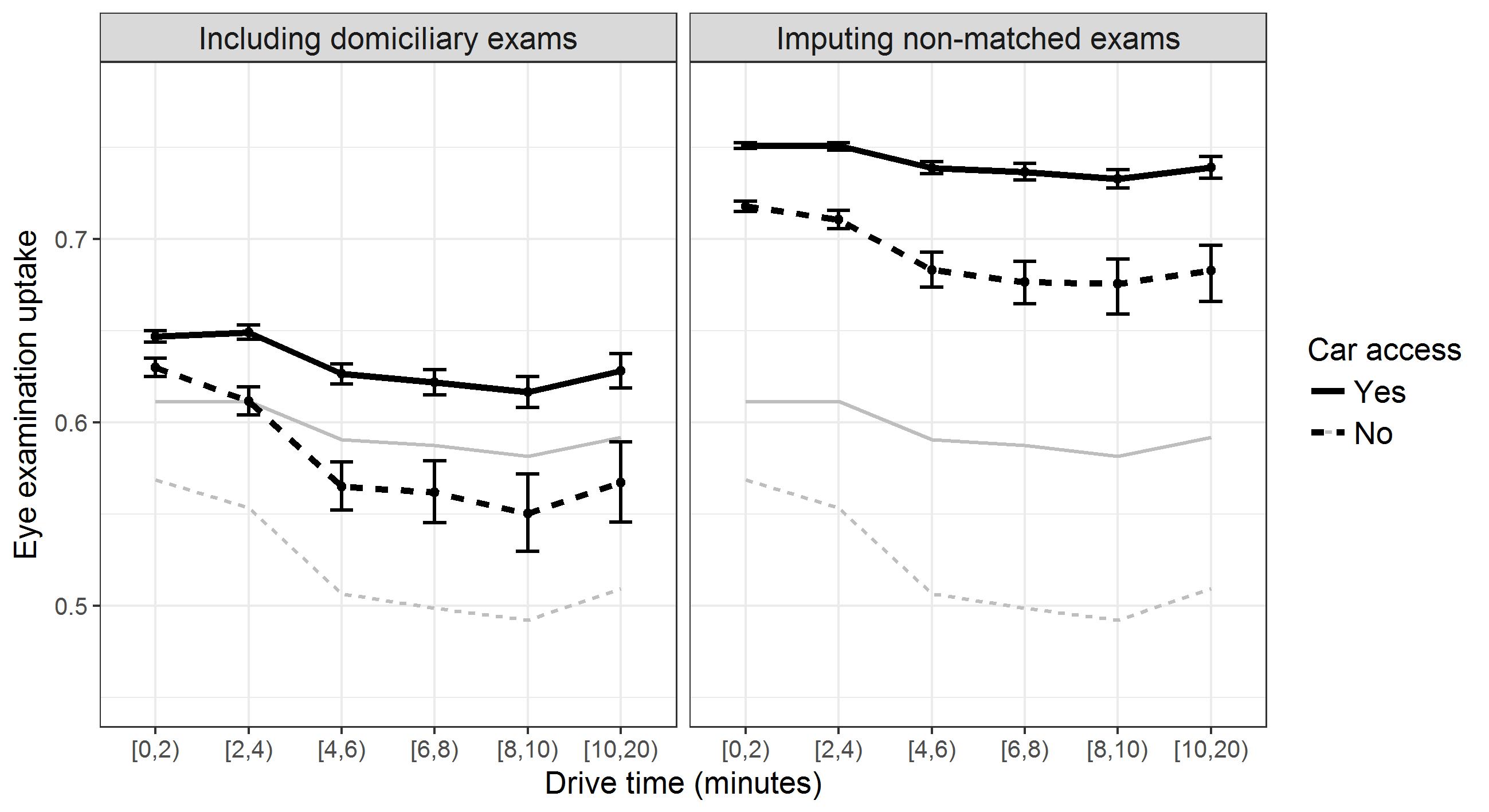

Supplement: Supplementary data [file bjophthalmol-2018-312201supp001.jpeg]
